# A Simple and Robust Single-Step Method for CAR-Vδ1 γδT Cell Expansion and Transduction for Cancer Immunotherapy

**DOI:** 10.3389/fimmu.2022.863155

**Published:** 2022-05-31

**Authors:** Gabrielle M. Ferry, Charles Agbuduwe, Megan Forrester, Sophie Dunlop, Kerry Chester, Jonathan Fisher, John Anderson, Marta Barisa

**Affiliations:** ^1^ Developmental Biology and Cancer Section, University Colloge of London (UCL) Great Ormond Street Institute of Child Health, London, United Kingdom; ^2^ TC-BioPharm, Holytown, United Kingdom; ^3^ Research Department of Oncology, Unicersity College of London (UCL) Cancer Institute, London, United Kingdom

**Keywords:** gamma delta (gammadelta) T cells, γδT cells, γδ T cells, V delta-1 (Vδ1) cells, CAR-gamma delta T cells, cancer immunotherapy, CAR-Vδ1 cells

## Abstract

The γδT cell subset of peripheral lymphocytes exhibits potent cancer antigen recognition independent of classical peptide MHC complexes, making it an attractive candidate for allogeneic cancer adoptive immunotherapy. The Vδ1-T cell receptor (TCR)-expressing subset of peripheral γδT cells has remained enigmatic compared to its more prevalent Vγ9Vδ2-TCR and αβ-TCR-expressing counterparts. It took until 2021 before a first patient was dosed with an allogeneic adoptive Vδ1 cell product despite pre-clinical promise for oncology indications stretching back to the 1980s. A contributing factor to the paucity of clinical progress with Vδ1 cells is the lack of robust, consistent and GMP-compatible expansion protocols. Herein we describe a reproducible one-step, clinically translatable protocol for Vδ1-γδT cell expansion from peripheral blood mononuclear cells (PBMCs), that is further compatible with high-efficiency gene engineering for immunotherapy purposes. Briefly, αβTCR- and CD56-depleted PBMC stimulation with known-in-the-art T cell stimulators, anti-CD3 mAb (clone: OKT-3) and IL-15, leads to robust Vδ1 cell expansion of high purity and innate-like anti-tumor efficacy. These Vδ1 cells can be virally transduced to express chimeric antigen receptors (CARs) using standard techniques, and the CAR-Vδ1 exhibit antigen-specific persistence, cytotoxicity and produce IFN-γ. Practicable, GMP-compatible engineered Vδ1 cell expansion methods will be crucial to the wide-spread clinical testing of these cells for oncology indications.

## Introduction

Characterized by expression of a T cell receptor (TCR) composed of gamma and delta chains (γδTCR), γδT cells are an innate-like subset of human T cells representing up to 15% of peripheral CD3-positive cells and up to 60% of intraepithelial lymphocytes in healthy donors. Whilst their physiological role in humans remains an area of active study and debate (1–3), γδT cells are a major area of interest in adoptive cell therapy for oncology indications (4–7). Exome characterisation of >16,000 patient tumors identified infiltrating γδT cells as the immune cell species most positively associated with patient survival across all cancers (8). A recent examination of patient brain tumor samples further defined γδT cell infiltration as most predictive of patient survival, in unexpected contrast to αβT cells, which correlated negatively with survival (9).

Two subsets composed of MHC-unrestricted Vγ9Vδ2-TCR and Vγ*x*Vδ1-TCR-expressing cells (where x denotes one of 6 functional gamma chain genes) dominate the peripheral γδT cell compartment. In contrast to the oligoclonal and phosphoantigen-reactive Vγ9Vδ2-TCR population, Vγ*x*Vδ1 cells (referred to hereafter as ‘Vδ1 cells’) express a Vδ1-TCR chain paired with one of various Vγ-chains. The peripheral human Vδ1 population has a polyclonal TCR repertoire that is reactive to a range of antigen types including peptides, lipids and various CD1 proteins of self and non-self origin (3, 10).

Adoptive transfer of the Vγ9Vδ2 cell subset has been clinically tested for anti-cancer efficacy for nearly 20 years (11). Several groups have also previously demonstrated methods to expand Vδ1 cells (12–18). 2021 saw publication of data from two first-in-man Vδ1 cell adoptive transfer clinical trials. GammaDelta Therapeutics Ltd are exploring unengineered, allogeneic ‘delta one T’ cell (‘DOT’; ‘GDX012’ product) safety, tolerability, and preliminary antileukemic activity in patients with minimal residual disease-positive acute myeloid leukemia (trial ID: NCT05001451). Adicet Bio Inc are testing the safety and efficacy of ‘ADI-001’ product anti-CD20 CAR-engineered allogeneic Vδ1 cells in adults with B cell malignancies, as a monotherapy or in combination with IL-2 (trial ID: NCT04735471). The discrepancy in the numbers of clinical investigations between Vδ1 and Vγ9Vδ2 γδT cell subsets does not stem from a lack of pre-clinical promise of the Vδ1 subset. Indeed, there is literature stretching back decades describing potent Vδ1 cell responses against tumor targets *in vitro* and graft-*versus*-leukemic effects following bone marrow transplantation, hypothesized to be mediated by atypical T cell subsets (19–22).

The discovery that Vγ9Vδ2 cells can be expanded to high numbers and purity using phosphoantigens (*e.g.* IPP or BrHPP) or phosphoantigen-inducing aminobisphophonates (*e.g.* zoledronic acid) enabled high-throughput Vγ9Vδ2 cell pre-clinical exploration, and consequently accelerated clinical translation. A promising two-step multi-cytokine clinical-grade protocol for Vδ1 cell expansion was published and patented by Almeida and colleagues in 2016 (23) (referred to hereafter as the ‘DOT protocol’) and is set for clinical translation in trial NCT05001451. Herein we describe a one-step, single-cytokine gene-engineered Vδ1 cell product manufacturing protocol that utilizes processes and reagents already employed to generate genetically modified αβT and Vγ9Vδ2 cell biotherapeutics. We show that Vδ1 cells are readily expandable to high numbers and purity by stimulation of αβTCR- and CD56-depleted PBMC with OKT-3 anti-CD3 mAb in the presence of IL-15-supplemented media. Thus-stimulated Vδ1 cells are efficiently and stably transduced with a chimeric antigen receptor (CAR) using standard viral transduction protocols. The resulting Vδ1-CAR-T cells exhibit innate recognition of targets in addition to antigen-specific boosting of function, and do not exhibit alloreactivity to allogeneic PBMC.

## Materials and Methods

### Ethical Approval

Expansion of T cells from healthy donors was performed under the governance of the following UCL UK research ethics committee approvals: “Establishing cell cultures for pediatric cancers”, IRAS project ID-154668. This ethical approval allows for expansion cell lines from tissue samples following written informed consent or from anonymized blood samples from healthy volunteers. For this study, only anonymized commercially available blood samples or anonymized small samples from healthy volunteers were used.

### γδT Cell Expansion

PBMC were isolated from purchased whole blood leucocyte cones *via* density gradient centrifugation using Lymphoprep (Stemcell) according to manufacturer’s instruction. PBMC were either cryopreserved in 90% FBS 10% DMSO or re-suspended in complete T cell culture media for further processing. Complete T cell culture media consisted of xeno- and serum-free CTS-OpTmizer (Thermo Fisher) with 10% synthetic serum replacement (Thermo Fisher) and GlutaMAX (Thermo Fisher), all of which are available to research as well as GMP-grade from Thermo Fisher with the following product catalogue numbers: research-grade CTS-OpTmizer (A1048501) and GMP-compatible alternative GMP-grade OpTmizer-CTS (A3705003), synthetic immune cell serum replacement that is compatible with both manufacturing standards (A2596101) and GlutaMAX also compatible with both standards (35050061). If starting with cryopreserved material, PBMC were thawed and rested at 10x10^6^ cells/mL in complete pre-warmed media overnight before further processing to avoid over-stressing the lymphocytes and to enhance depletion quality. PBMC at 2-4x10^6^ cells/mL density were then either stimulated in standard cell culture plates right away or first depleted of αβT cells using the TCRα/β Product Line (Miltenyi Biotec) according to manufacturer’s instructions concurrently with depletion of CD56-positive cells using CD56 MicroBeads (Miltenyi Biotec) according to manufacturer’s instructions. Briefly, cells were first labelled with anti-TCRα/β-biotin, then a mix of anti-biotin microbeads and anti-CD56 beads, and then depleted using MACS Cell Separation LD Columns (Miltenyi Biotec). If cultured in G-Rex vessels (Wilson Wolf), depleted PBMC were initiated at 2-4x10^6^ cells/cm^2^. Thus-prepared PBMC were stimulated with either 1μg/mL OKT-3 (Miltenyi Biotec Cat# 130-093-387, RRID : AB_1036144) or 1μg/mL PHA (Merck) and various cytokine combinations: (i) 100 IU/mL IL-2 aldesleukin (Proleukin; Novartis), (ii) 70 ng/mL IL-15 (Peprotech), (iii) 20 ng/mL rhIL-7 (Peprotech), or the (iv) ‘DOT protocol’ cytokine cocktail, which consisted of a first culture in 100 ng/mL rIL-4, 70 ng/mL rIFN-γ, 7 ng/mL rIL-21 and 15 ng/mL rIL-1β followed by a second culture in 70 ng/mL rIL-15 and 30 ng/mL IFN-γ (all from Peprotech). When comparing the full ‘DOT protocol’ to test expansion protocols, the methodology described by Almeida and colleagues was used (23), albeit with the omission of a positive selection step using OKT-3 following the alpha beta TCR depletion. Briefly, depleted PBMC were stimulated for a first cytokine culture with 70ng/mL OKT-3, and then a second cytokine culture with 1μg/mL OKT-3. Live cells before and during expansion were counted using Trypan Blue exclusion, an automatic cell counter (Invitrogen) and flow cytometry-based Precision Count Beads (Biolegend).

### Vδ2 γδT Cell Depletion

Vδ2 γδT cells were depleted from PBMC at one of three stages of expansion: pre-initiation, at midway split or at harvest. All depletions were done using anti-TCR/Vδ2 mAb clone B6 (BioLegend Cat# 331404, RRID : AB_1089228) at a concentration of 0.5µg/10^6^ PBMC. When depleting at initiation Vδ2 cell initiation was incorporated into the αβTCR/CD56 depletion process. This was done as follows: PBMC were co-incubated with αβTCR-biotin mAb and Vδ2 (clone: B6)-biotin mAb, washed, and then co-incubated with anti-biotin and anti-CD56 microbeads according to manufacturer’s protocol, then washed and depleted using Miltenyi LD magnetic column separation, as above and according to manufacturer’s protocol. If depleting at midway split or final harvest, expanding cells were harvested, washed and labelled with 0.5µg clone B6/10^6^ PBMC, incubated for 20min, washed and incubated and depleted using Miltenyi anti-biotin microbeads and LD columns as above.

### Flow Cytometry

The following fluorochrome-antibody conjugates and dyes were used according to manufacturer’s instruction in Biolegend Cell Staining Buffer to detect different lymphocyte subpopulations in culture: Zombie Green Viability Dye (BioLegend), Zombie Yellow Viability Dye (BioLegend), LIVE/DEAD Fixable Near IR kit (Thermo Fisher), anti-CD3 PE/Dazzle594 (BioLegend Cat# 980006, RRID : AB_2715768), anti-αβTCR APC (BioLegend Cat# 306717, RRID : AB_10612747), anti-TCRVδ1 APC-Vio770 (Miltenyi Biotec Cat# 130-120-440, RRID : AB_2752099), anti-TCRVδ2 VioBlue and PE (Miltenyi Biotec Cat# 130-101-152, RRID : AB_2660779), anti-CD69 FITC (BioLegend Cat# 310903, RRID : AB_314838), anti-NKG2D PercP/Cy5.5 (BioLegend Cat# 320817, RRID : AB_2562791) anti-CD56 Alexa Fluor 488 (BioLegend Cat# 318311, RRID : AB_604094), anti-PD-1 APC/Fire750 (BioLegend Cat# 329953, RRID : AB_2616720) and BUV737 (BD Biosciences Cat# 612791, RRID : AB_2870118), anti-LAG-3 PE/Cy7 (BioLegend Cat# 369309, RRID : AB_2629752), anti-TIM-3 BV711 (BioLegend Cat# 345023, RRID : AB_2564045), anti-CD34 QBend10 Alexa Fluor700 (BioTechne), anti-CD34 QBend10 Alexa Fluor488 (Novus Biologicals), anti-NKp44 PerCP/Cy5.5 (BioLegend Cat# 325114, RRID : AB_2616752), anti-NKp30 DyLight 650 (NovusBio, Cat # FAB1849W, clone 210845). When detecting intracellular and cell surface accumulation of IFN-γ and CD107a, respectively, PBMC were challenged with relevant targets overnight, and incubated at 37°C and 5% CO_2_ with anti-CD107a FITC (BioLegend Cat# 328605, RRID : AB_1186058), then 1x monensin (Biolegend) was added followed by incubation for another 4h, stained for cell surface markers, and then permeabilized using Biolegend Intracellular Staining Permeabilization Wash Buffer and stained with anti-IFN-γ Brilliant Violet 605 (BioLegend Cat# 502535, RRID : AB_11125368), anti-IL17a PerCPCy5.5 (BioLegend Cat# 512313, RRID : AB_961397) or anti-Granzyme B Pacific Blue (BioLegend Cat# 372217, RRID : AB_2728384) according to manufacturer’s instructions. All expansion and activation samples were analyzed on a BD LSR II flow cytometer using FACSDiva software (BD FACSDiva Software, RRID : SCR_001456), while CAR-Vδ1 proliferation was analyzed on a Beckman Coulter CytoFlex using CytExpert software (CytExpert Software, RRID : SCR_017217). For setting of gates in analysis of panels we employed fluorescence minus one (FMO) controls. Post-acquisition data processing was carried out using FlowJo software (FlowJo, RRID : SCR_008520). T-SNE analysis on flow cytometry data was performed using FlowJo software and concatenated using R language Statistical Computing (RRID : SCR_001905).

### Retroviral Production and T Cell Transduction

293T cells (ATCC Cat# CRL-3216, RRID : CVCL_0063) were plated at 1.5x10^6^ cells per 10cm^2^ plate (Corning) in 10mL 10% fetal bovine serum (FBS)-supplemented Gibco IMDM (Thermo Fisher). At 70% confluence, 293T cells were transfected using GeneJuice (Merck) according to manufacturer’s protocol. Triple plasmid transient transfection was carried out using SFG-gammaretroviral vectors (RRID : Addgene_22493). The anti B7H3 CAR-T was synthesized within SFG and contains the following components: IL-2 signal peptide, TE9 ScFv, CD8 hinge and transmembrane, CD28 endodomain, CD3zeta. The CAR was co-expressed with the RQR8 sort suicide gene allowing detection with anti-CD34 antibody.

The B7H3-CAR (second generation with CD28 and 325 CD3-zeta endomains synthesized in our laboratory), gag+pol 326 (RRID : Addgene_8449) and RD114 envelope (RRID : 327 Addgene_17576) plasmids were added at an equimolar ratio. Retroviral supernatant was harvested at 48 and 72 hours following transfection and used immediately for T cell transduction. Briefly, non-tissue culture treated 24 well plates (Costar) were coated with RetroNectin (Takara) in PBS (final concentration of 1mg/mL) and incubated at 4°C for 24 hours. The retronectin was removed and 1.5 mL of retroviral supernatant was added to each retronectin coated well. Following this, 3x10^5^ stimulated T cells in 500 µL was added and plates were centrifuged at 1000 *x* g for 40 minutes, at room temperature before incubation in complete T cell culture media at 37°C, supplemented with IL-15 to a final concentration of 70ng/mL (~140 IU/mL). Transduced T cells were harvested after three days, washed and re-suspended for expansion in specified cytokine-supplemented complete T cell culture medium. Transduction efficiency was assessed by flow cytometric detection of the CD34 marker gene (26).

### Cytotoxicity Assays

Cytotoxicity was determined either by staining for cell surface accumulation of CD107a as above where indicated, or by four-hour chromium (^51^Cr)-release assay. Briefly, 1x10^6^ target cells were labelled with 20 µL ^51^Cr amounting to 3.7 MBq (PerkinElmer) for 60 minutes at 37°C. Following this, target cells were co-cultured with effector CAR T cells at range of effector: target (E:T) ratios (10:1, 5:1, 2.5:1 and 1.25:1) for four hours at 37°C in 96 well U bottom plates (Grenier). After incubation, the plates were centrifuged at 1500RPM for 5 minutes and 50 µL of the supernatant was transferred to 96 well OptiPlate-96 HB (PerkinElmer). 150 µL of scintillation fluid was added per well and the plates were sealed and incubated at room temperature overnight. ^51^Cr release from lysed target cells was counted on 1450 MicroBeta Trilux Scintillation Counter (PerkinElmer). The scintillation counts from wells with only targets (without effectors) were used as spontaneous release controls and target cells lysed with 1% Triton X-100 (Thermofisher) were used as a maximum ^51^Cr release control.

### Proliferation Assay

Proliferation of expanded and harvested Vδ1 cells following repeated stimulation was evaluated to determine CAR-Vδ1 persistence in the presence of an antigen-expressing target cell line. Briefly, CAR-Vδ1 cells were labelled with CellTrace Violet proliferation dye (ThermoFisher) according to manufacturer’s instructions for 20 minutes at 37°C. Once labelled, CAR-Vδ1 cells were plated at 5x10^5^ per well of a 48-well plate (Corning) and co-cultured at a 1:1 E:T ratio with irradiated tumor targets, either B7H3-negative Jurkat wild type cells (Jurkat-WT) or isogenic Jurkat cells transduced to express high levels of B7H3 (Jurkat-B7H3). Plates were incubated at 37°C and 5% CO_2_ for 6 days, without exogenous cytokine supplementation. Freshly irradiated target cells were fed every two days following co-culture and proliferation was evaluated by flow cytometry.

### Cell Lines

Jurkat (ATCC Cat# TIB-152, RRID : CVCL_0367), HeLa (ATCC Cat# CCL-2.2, RRID : CVCL_0058), NOMO-1 (DSMZ Cat# ACC-542, RRID : CVCL_1609), K562 (ATCC Cat# CCL-243, RRID : CVCL_0004) and U87 (ATCC Cat# HTB-14, RRID : CVCL_0022) cell lines were all acquired from ATCC and cultured as recommended by the supplier. LAN-1 cell line (DSMZ Cat# ACC-655, RRID : CVCL_1827) was acquired from DSMZ and cultured as recommended by the supplier. Cell lines were screened monthly for mycoplasma contamination Briefly, Jurkat, K562 and NOMO-1 cells were grown in 10% FBS-supplemented RPMI1640 (Sigma Aldrich) suspension culture and kept at <1x10^6^/mL density. LAN-1, HeLa and U87 cell lines were grown in 10% FBS-supplemented DMEM (Thermo Fisher) adherent culture and split regularly at around 80-90% confluency using trypsin (Thermo Fisher)-based disaggregation, to avoid overgrowth.

### Production of B7-H3 Positive Jurkat Cells

A truncated B7-H3 (T-B7-H3) in an SFG γ-retroviral expression cassette was a gift from Karin Straathof (UCL). A 4Ig-B7-H3 isoform of B7-H3 was purchased (Sinobiological) and cloned into a γ-retroviral expression cassette. Retroviral transduction was used to stably transduce Jurkat cells with 4Ig-B7-H3.

### Statistical Design

Data was analyzed with GraphPad Prism (GraphPad Prism, RRID : SCR_002798). Data are displayed at mean ± SEM unless otherwise stated. For normally distributed numerical data, parametric tests were used to determine significance of difference between groups. Analysis of variance (ANOVA) was used, unless otherwise stated. Significance is represented by: *p<0.5, **P<0.01, ***p<0.001, ****p<0.0001.

### Calculation of Earth Mover’s Distance (EMD)

EMD describes change in signal strength based on difference in probability distribution, with a higher EMD denoting a larger change. The use of EMD to describe changes in protein accumulation allows multiple biological replicates to be characterized with a high degree of consistency, without collapsing the data to mean or median values at the expense of interpretability (24). EMD was computed between bulk CAR-transduced T cell culture versus non-transduced culture. Samples were time- and donor-matched. EMD was calculated between T cell populations that had undergone the same processing. The Python (Python Programming Language, RRID : SCR_008394) module ‘wasserstein_distance’, which is a component of ‘scipy.stats’, was used to calculate EMD between samples.

## Results

### OKT-3 and IL-15 Stimulation of αβTCR- and CD56-Depleted PBMC Leads to Robust and Reproducible Vδ1 Cell Expansion

**Figure 1:**
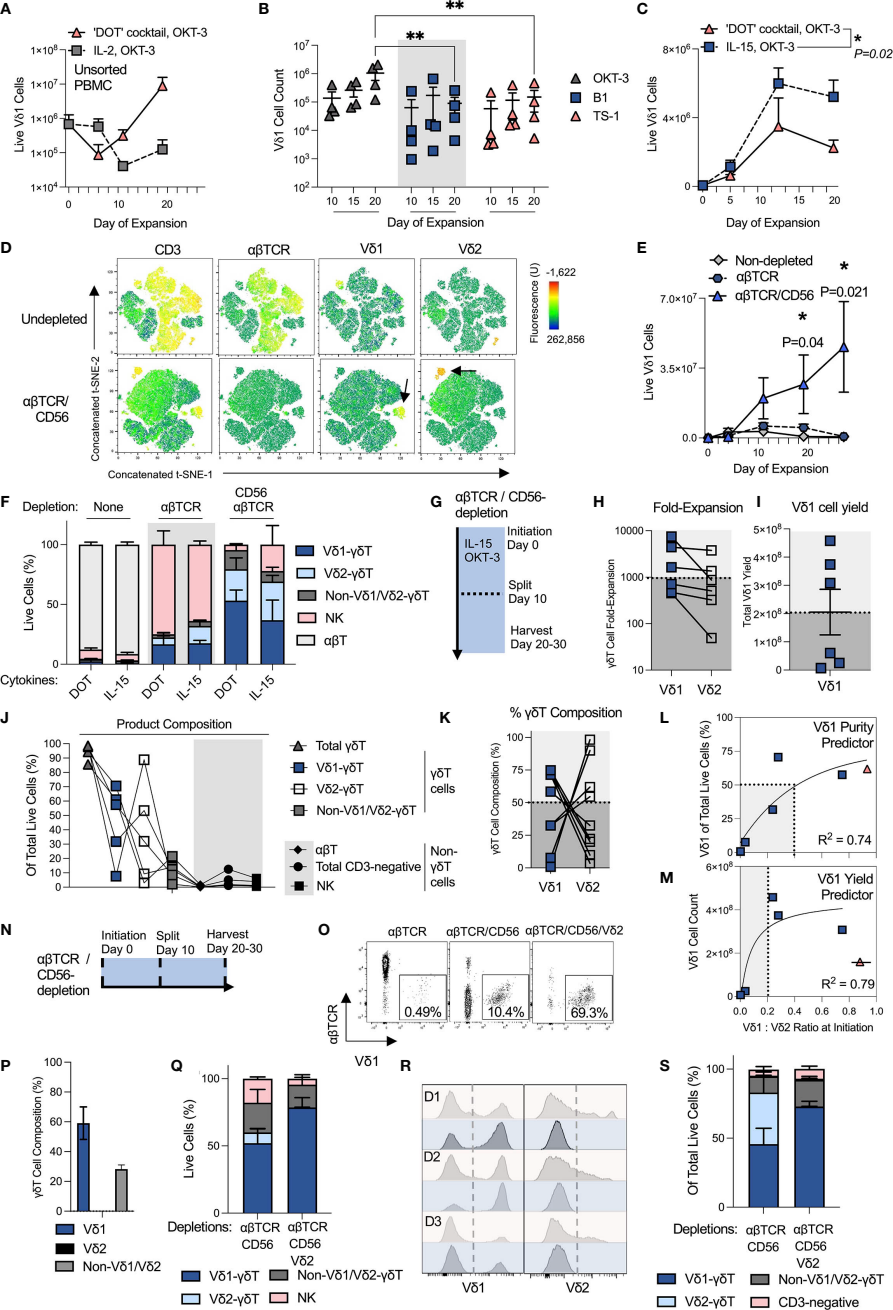
OKT-3 and IL-15 stimulation of αβTCR- and CD56-depleted PBMC leads to robust and reproducible Vδ1 cell expansion. All experiments used separate and independent donors except where indicated they were from the same donors. **(A)** Vδ1 cell yield was compared when expanding PBMC with either the ‘DOT’ cocktail of cytokines or IL-2 (N=2; mean +/- standard error mean (SEM). **(B)** αβT-depleted PBMC were expanded with the ‘DOT’ cocktail of cytokines and a single stimulation with 1ug/mL of either anti-CD3 mAb clone OKT-3, anti-γδTCR mAb clone B1 or Vδ1-TCR clone TS-1. (N=4; mean +/- SEM; statistical significance was determined by two-way ANOVA with Sidak’s multiple comparisons test). **(C)** αβT-depleted PBMC were expanded with either the ‘DOT’ cocktail of cytokines or IL-15. Expansion was measured over a period of 20 days, and compared at day 20 post-stimulation (N=3; mean +/- SEM; statistical significance was determined by two-way ANOVA with Sidak’s multiple comparisons test). Vδ1 cell numbers in the starting material ranged from 40e3 – 80e3/well. **(D)** Three separate donor PBMC were stained for flow cytometry analysis of population composition before and after αβTCR/CD56 depletion and visualized using a t-SNE algorithm. The three donor t-SNE data was concatenated using ‘R’. The black arrows on the bottom row indicate Vδ1 and Vδ2 cells among the depleted PBMC. **(E)** Vδ1 cells were expanded using OKT-3 and IL-15 from PBMC either unmanipulated, depleted only of αβT cells, or depleted of αβT cells and CD56-positive cells (N=3; mean +/- SEM; statistical significance was determined by ordinary two-way ANOVA with Sidak’s multiple comparisons test). Vδ1 cell numbers in the starting material ranged from 7e3 – 80e3/well. **(F)** Day 20-harvested expansate composition was compared after culture in either the ‘DOT’ cocktail of cytokines or IL-15 alone, from either undepleted, αβTCR single-depleted or αβTCR/CD56-double depleted PBMC at initiation (N=3). **(G)** To achieve optimal 20 day expansion, expanding T cell cultures were split 1:4 at day 10 of expansion to avoid overconfluence. **(H)** Matched Vδ1 cell expansions using OKT-3 and IL-15 from αβTCR/CD56-depleted PBMC in 6-well G-Rex vessels were compiled from six different donors and two separate experimental repeats and compared in terms of fold-expansion and **(I)** absolute cell yield. **(J)** A further five donors were initiated for G-Rex culture in a third αβTCR/CD56-depleted OKT-3/IL-15 manufacturing run. Day 20 expansates were analyzed for product composition (N=5). **(K)** G-Rex expansion donor data was pooled to examine the Vδ1/Vδ2 cell composition of total product γδT cells (N=11). **(L)** The effect on αβTCR/CD56-depleted IL-15/OKT-3-expanded Vδ1 cell purity of pre-depleted, pre-stimulation PBMC Vδ1:Vδ2 ratio was determined (N=6; statistical significance was determined by a non-linear least squares model). Purity in this context denotes Vδ1 cells of total live cells in the product at day 20. **(M)** The effect on αβTCR/CD56-depleted IL-15/OKT-3-expanded Vδ1 cell yield of pre-depleted, pre-stimulation PBMC Vδ1:Vδ2 ratio was determined (N=6; statistical significance was determined by a non-linear least squares model; one data point indicated in red and crossed out was not included in the statistical analysis of the data). **(N)** Three potential timepoints (shown with black, dotted lines) were identified for testing Vδ2 cell depletion from Vδ1 cell product: at initiation, at midway split or at harvest. **(O)** Initiation Vδ2 depletion: Vδ1 cell purity of total T cells (CD3-positive cells) were compared in single (αβTCR), double (αβTCR/CD56) or triple (αβTCR/CD56/Vδ2)-depleted freshly-isolated PBMC at initiation. Shown are representative dot plots from one donor. **(P)** Initiation Vδ2 depletion: Three donor triple αβTCR/CD56/Vδ2-depleted PBMC OKT-3/IL-15 expansates were harvested at day 10 of expansion to examine γδT cell subset composition (N=3). **(Q)** Midway Vδ2 depletion: αβTCR/CD56-depleted PBMC were expanded with OKT-3/IL-15, then depleted of Vδ2 cells at day 10 and plated for another 10 days’ expansion until day 20. Resulting day 20 PBMC composition was analyzed and compared in Vδ2-depleted (αβTCR/CD56/Vδ2) or undepleted (αβTCR/CD56) expansates (N=3). **(R)** Harvest Vδ2 depletion: Expansate depletion of Vδ2 cells was tested at day 20 harvest of cell cultures. Shown are three donor (D1, D2, D3) PBMC pre- (red) and post- (blue) depletion, in which Vδ1 and Vδ2 cells was measured (N=3). **(S)** Harvest Vδ2 depletion: Expansate product composition was characterized following day 20 product Vδ2-depletion (N=3). * means P <0.05; ** means P ≤0.01.

Benchmarking our efforts to the two-step and multi-cytokine ‘DOT protocol’ [described in detail by Almeida and co-workers (23)], we compared its ability to expand Vδ1 cells with canonical *ex vivo* T cell expansion methodology, consisting of a single step PBMC stimulation with clone OKT-3 anti-CD3 mAb and IL-2 at 100 IU/mL. Briefly, the ‘DOT protocol’ entails a first 10 day culture in OKT-3 with IL-4, IFN-γ, IL-21 and IL-1β, followed by a second culture in OKT-3 with IL-15 and IFN-γ. Over 20 days of expansion, the ‘DOT’ cocktail of cytokines yielded a mean 100-times more Vδ1 cells than culture in IL-2 following activation of unsorted PBMC with a single dose of OKT-3 at initiation (**Figure 1A**). The inferiority of IL-2 monoculture to the ‘DOT’ cocktail of cytokines is consistent with what was reported in the original ‘DOT’ protocol publication (23). In pursuit of an allogeneically-applicable expansion protocol that generates a product without potentially alloreactive αβT cells, we then depleted the starting PBMC of αβT cells using a standard and GMP-compatible αβTCR-biotin and anti-biotin bead-based protocol from Miltenyi. Aside from removing contaminating αβT cells, αβTCR-depletion further enhanced the yield of Vδ1 cells following 20 day culture in the ‘DOT’ cocktail of cytokines with a single dose of OKT-3 at initiation (**Supplementary Figures 1A, B**). This may be at least in part be due to the removal of αβT cell competition for cytokines.

We then investigated, in the context of the DOT cocktail of cytokines, whether we could expand Vδ1 cells more efficiently by using a more specific γδTCR stimulus, such as an anti-γδTCR mAb (clone: B1) or specific anti-Vδ1-TCR mAb (clone: TS-1). A single stimulating 1 μg/mL mAb dose at initiation has been previously reported to be effective for TS-1/B1 mAb-driven Vδ1 cell expansion (25). Anti-CD3 OKT-3 stimulation led to an order of magnitude higher Vδ1 cell expansion from non-depleted PBMC than either γδT cell-specific clone (**Figure 1B**). Moreover, specific anti-γδTCR stimulation applied to non-depleted PBMC failed to prevent expansion of contaminating αβT cells in culture (**Supplementary Figure 1C**). Vδ1 cell numbers in DOT cytokine cocktail culture were evaluated and found equivalent between single stimulation with OKT-3 at initiation or repeated OKT-3 stimulation at 5 day intervals over the course of expansion, suggesting that a single OKT-3 administration is sufficient for optimal T cell expansion (**Supplementary Figure 1D**). As a result, αβTCR-depletion and a single stimulation with OKT-3 anti-CD3 mAb were progressed for further study.

We next evaluated Vδ1 cell expansion in this culture setup using either the two-step DOT cocktail of cytokines or continuous culture in 70ng/mL (corresponding to ~140 IU/mL) of IL-15 alone, it also being a component of the latter half of the DOT protocol regimen. Having discarded IL-2 alone as an optimal milieu, IL-15 was chosen as the second most commonly-employed GMP-compatible T cell manufacturing mitogen. Unexpectedly, IL-15 monoculture yielded at least equivalent or higher Vδ1 cell numbers to the DOT cocktail of cytokines (**Figure 1C**). We next examined whether IL-15-driven Vδ1 cell expansion could be improved by further depleting competition for cytokine from NK cells. We first confirmed that freshly-isolated PBMC Vδ1 cells do not express canonical NK cell-marker, CD56, while CD3-negative freshly-isolated PBMC and some Vδ2 cells do (**Supplementary Figure 1E**). We then combined the αβTCR-biotin and anti-biotin depletion step with GMP-compatible Miltenyi anti-CD56 magnetic beads according to manufacturer’s protocol. Three donor concatenated t-SNE analysis of culture initiation material demonstrates the difference between undepleted and αβTCR/CD56-depleted freshly-isolated PBMC (**Figure 1D**). The double-depleted material is predominantly CD3-negative, though with enriched Vδ1 and Vδ2 composition relative to undepleted PBMC.

Undepleted, αβTCR- and αβTCR/CD56-depleted PBMC starting material was then compared for its ability to expand Vδ1 cells when stimulated with IL-15 and OKT-3. Double-depleted PBMC yielded not only substantially greater Vδ1 cell numbers, but also purity (**Figures 1E, F**). Of note, no substantive differences in product composition from any of the starting materials could be found when comparing IL-15 monoculture with culture in the DOT cocktail of cytokines (**Figure 1F**). We, therefore, progressed a single-step OKT-3 + IL-15-based αβTCR/CD56-depleted Vδ1 expansion protocol for further optimization. We note that in this setup, a majority of donor αβTCR/CD56-depleted PBMC initially plated at 1x10^6^ cells/cm2 approached over-confluence by day 10 of culture (**Supplementary Figure 1F**). We, therefore, opted for a 1:4 culture split midway through the protocol (**Figure 1G**). While feasibly the cells can be cultured for shorter or longer periods as per desired product specification, we progressed a 20-day expansion period with a midway split for further analysis.

We benchmarked our OKT-3 and IL-15-based single step protocol against other published single step Vδ1 expansion methods that utilize phytohaemagglutinin (PHA) instead of anti-CD3 mAb (16–18). We stimulated αβTCR- and CD56-depleted PBMC with OKT-3 and IL-15, or PHA with either IL-2 (16, 17) or IL-7 (18). OKT-3 with IL-15 outperformed both PHA-based protocols in terms of Vδ1 yield in all donors tested (**Supplementary Figures 2A-D**). The choice of anti-CD3 stimulation was further re-enforced by data indicating that, at harvest, OKT-3-stimulated Vδ1 cells expressed higher activation marker levels with concurrently lower exhaustion markers than PHA-stimulated Vδ1 cells, all the while expressing more NKG2D and CD56 receptors, indicative of favorable functional phenotype (**Supplementary Figure 2E**). Indeed, PHA-stimulated Vδ1 cells expressed higher apoptotic markers than CD3-stimulated Vδ1 cells at harvest (**Supplementary Figure 2F**).

Encouragingly for clinical practicality, OKT-3 with IL-15 expanded not only freshly-isolated PBMC, but also Vδ1 cells from thawed cryopreserved PBMC that were αβTCR-/CD56-depleted following an overnight rest upon resuscitation (**Supplementary Figures 3A, B**). We note that an overnight PBMC ‘rest and recovery’ step in complete media at standard culture conditions enabled retention of a pre-cryopreservation Vδ1/Vδ2 cell ratio (**Supplementary Figure 3C**), and substantially increased the quality of αβTCR-/CD56-depletions as well as Vδ1 cell expansion. Resting was carried out at a high cell density (10^6^ PBMC/mL) in complete expansion media, without cytokine supplementation.

To simulate a potential manufacturing process, we compiled expansion data of six arbitrarily chosen healthy donor cryopreserved leukapheresate-derived PBMC from two experimental runs, each of which consisted of PBMC thaw and overnight rest in complete media, followed by αβTCR/CD56-depletion and OKT-3/IL-15 stimulation the following day, as described above, except that in this iteration Vδ1 cells were cultured in 6-well G-Rex (as opposed to standard cell culture) vessels. Expansions were split into new wells at a 1:4 culture surface area ratio on day 10 of expansion and harvested at day 20 for analysis. Out of six donors tested, three achieved >1,000-fold Vδ1 cell expansion, and all achieved >400-fold expansion (**Figure 1H**). While in every donor examined Vδ1 cell expansion rate was greater than that of Vδ2 cells, in five out of six donors the difference was minimal suggesting a relatively unbiased γδT subset expansion by OKT-3/IL-15 (**Figure 1H**). The total Vδ1 cell yield per harvested 6-well G-Rex well was >2x10^8^ Vδ1 cells per 4x10^6^ PBMC initiated in three out of six donors tested, delineating apparent ‘good’ and ‘poor’ expanders (**Figure 1I**). These donors further clustered by product composition. While all yielded 80-100% pure γδT cells, γδT cell composition varied greatly (**Figure 1J**). αβT cell contamination was negligible, though some CD3-negative cells (mostly NK cells) persisted (**Figure 1J**). We investigated γδT cell product composition further and found that in all G-Rex-expanded donor products, an apparently inverse relationship existed between high purity Vδ1-donors and Vδ2-donors (**Figure 1K**). Given the relatively unbiased subset expansion by OKT-3/IL-15 we observed, we interrogated whether a high-purity (or ‘good’) Vδ1-donor could be predicted by examining the undepleted leukapheresates of donors entered for expansion.

The pre-depletion donor PBMC Vδ1:Vδ2 ratios were compared in six donors and correlated to Vδ1 cell purity and total Vδ1 cell count after 20 day stimulation of αβTCR/CD56-depleted PBMC with OKT-3 and IL-15. In these donors, a pre-initiation Vδ1:Vδ2 ratio of greater than 0.4:1 was associated with at least 50% Vδ1 cell purity at harvest (R^2 =^ 0.74) (**Figure 1L**). The relationship between Vδ1:Vδ2 ratio and absolute Vδ1 cell yield was also investigated. We observed, however, that with excluding one donor from analysis for yield (indicated in red in **Figures 1L, M**), high Vδ1:Vδ2 ratio at initiation correlated with high Vδ1 cell yield at harvest in this small sampling of independent donors. A minimum pre-initiation Vδ1:Vδ2 ratio of 0.2:1 was associated with harvests of >2x10^8^ Vδ1 cells per 4x10^6^ PBMC initiated (R^2 =^ 0.79) (**Figure 1M**). We hypothesize, therefore, that a high Vδ1:Vδ2 ratio at initiation of expansion may serve as a biomarker for high ultimate Vδ1 cell yield and purity at harvest, though more donor material screening is required to substantiate this observation.

We next examined whether αβTCR/CD56-depleted OKT-3/IL-15-stimulated product can be further enriched for Vδ1 cells by depleting contaminating Vδ2 cells. To this end, we identified three potential depletion points during the manufacture: at initiation concurrently with αβTCR/CD56-depletion, midway at the split, or at harvest (**Figure 1N**). First examining the initiation depletion strategy, we compared the following depletions for Vδ1 cell purity among freshly-isolated PBMS, (i) αβTCR, (ii) αβTCR/CD56, and (iii) αβTCR/CD56/Vδ2. The triple depletion was performed as follows: PBMC were co-incubated with αβTCR-biotin mAb and Vδ2 (clone: B6)-biotin mAb, washed, and then co-incubated with anti-biotin and anti-CD56 microbeads according to manufacturer’s protocol. Each depletion step further increased Vδ1 cell purity among the initiation T cell compartment (**Figure 1O**). Not only was the clone B6 Vδ2-depletion highly effective at the outset, it also prevented re-growth of Vδ2 cells during expansion (**Figure 1P**). Though, it also encouraged non-Vδ1/Vδ2 γδT cell expansion, resulting in a Vδ1: non-Vδ1/Vδ2 γδT cell ratio of ~ 2:1. Product Vδ2 depletion midway was highly efficacious; at harvest it yielded a ~77% pure Vδ1 cell product, with a ~17% non-Vδ1/Vδ2 γδT cell presence (**Figure 1Q**). Product depletion at harvest yielded a similar purity of ~72% Vδ1 cells and ~20% non-Vδ1/Vδ2 γδT cells (**Figures 1R, S**). Non-γδT cell content was ~10% in all methods tested and was largely CD3-negative. A majority of these were NK cells (CD3-negative/CD56-positive PBMC) (**Supplementary Figure 4B**), that we hypothesize either escaped initial depletion or upregulated CD56 during expansion. We note that, while initially negative, also 50-70% of Vδ1 cells upregulated CD56 upon expansion (**Supplementary Figure 4C**), negating the possibility of a CD56-based contaminant depletion at harvest.

Of the Vδ2 cell depletion strategies tested, we hesitate to recommend the best, nor indeed whether it is required at all - as optimal product specifications in terms of γδT cell subset purity for maximal therapeutic efficacy are yet to be determined. It is not necessarily the case that the purest Vδ1 cell product is the most efficacious against cancer, and it is feasible that other γδT cell subsets in the product will synergize rather than suppress Vδ1 cell anti-cancer functionality. Substantial further study in this area is required. Other factors that will impact the decision on Vδ2 cell depletion include post-harvest processing, such as intention to cryopreserve, *etc.* The remainder of the functional data in this study is presented on Vδ1 cell products derived from double (αβTCR/CD56)-depleted PBMC.

We note that the above depletions could be reproduced to GMP-standard by replacing research-grade αβTCR and CD56 depletion reagents with GMP-grade alternatives from Miltenyi Biotec (CliniMACS TCRα/β Product Line cat nr: 200-070-407; CliniMACS CD56 Product Line cat nr: 170-076-713) and carried out on either CliniMACS *Plus* or CliniMACS *Prodigy* hardware. We note the lack of commercially-available GMP-compatible clone B6 Vδ2-biotin products, though anticipate that those could be obtained from suppliers through custom manufacture.

### OKT-3/IL-15-Expanded Vδ1 Cells Are Innately Cytotoxic Against a Range of Tumor Targets

**Figure 2:**
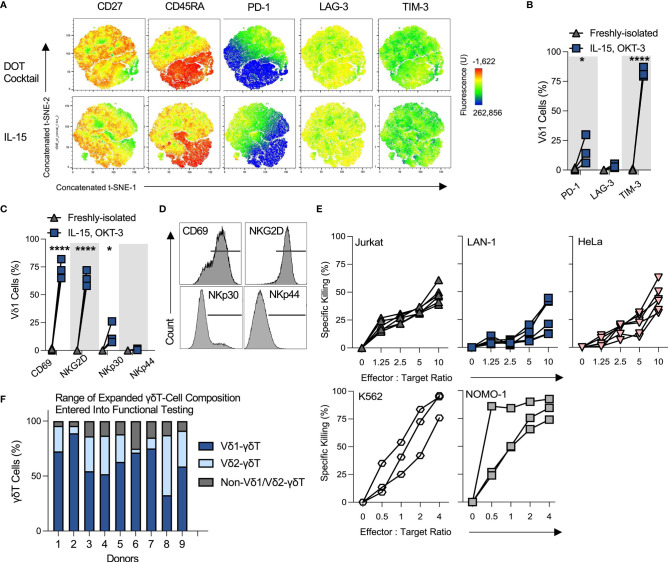
OKT-3/IL-15-expanded Vδ1 cells are innately cytotoxic against a range of tumor targets. All the following data describes phenotyping and performance of Vδ1 cells expanded using OKT-3 and IL-15 from αβT and CD56-depleted PBMC, unless specified otherwise. **(A)** Three donor day 20-expanded Vδ1 cell expression of a range of memory and exhaustion markers was analyzed using t-SNE and concatenated. Profiles were compared between IL-15 or ‘DOT’ cytokine cocktail milieus (N=3). **(B)** Vδ1 cell % positivity of ‘exhaustion’ markers was tallied in three donors (N=3; statistical significance was ascertained using a matched two-way ANOVA with Sidak’s multiple comparison test). **(C)** Activation and cytotoxicity markers CD69, NKG2D, NKp30 and NKp44 were analyzed using flow cytometry (N=3; statistical significance ascertained using a matched two-way ANOVA with Sidak’s multiple comparison test), **(D)** with histograms of marker expression shown from a representative donor. **(E)** Expanded Vδ1 cell product cytotoxicity was studied by target chromium (Cr^51^) release upon 4h co-culture at different E:T ratios (N=6; each dot and trajectory represent a single donor) for Jurkat, LAN-1 and HeLa target cell lines, and by use of an overnight flow cytometric cytotoxicity assay for K562 and NOMO-1 target cell lines (N=3). **(F)** Donors for functional assay testing were not selected based on Vδ1 cell purity. Shown is a representative range of 9 donor day 20 OKT-3/IL-15-expanded product γδT cell composition from αβTCR/CD56-depleted PBMC (N=9). * means *P* < 0.05; **** means *P* ≤ 0.0001.

20-day-expanded OKT-3/IL-15 Vδ1 cells exhibited a similar memory and exhaustion profile to DOT cytokine cocktail counterparts. As indicated in concatenated t-SNE plots of various markers, Vδ1 cells were broadly positive for CD27, with a subpopulation brightly expressing CD45RA. While a proportion of CD27+/CD45RA- cells expressed PD-1, few Vδ1 cells bound anti-LAG-3 antibody above isotype control (**Figures 2A, B**). Nearly all Vδ1 cells were dimly but universally TIM-3-positive. OKT-3/IL-15-expanded Vδ1 cells further upregulated activation marker CD69 as well as cytotoxic differentiation marker NKG2D, but not NKp44 (**Figures 2C, D**). A small subpopulation of expanded Vδ1 cells consistently upregulated NKp30.

Functionally, OKT-3/IL-15-expanded Vδ1 cells exhibited highly consistent innate cytotoxicity against a range of hematological and solid tumor targets, including T cell leukemia Jurkat cells, cervical cancer HeLa cells, neuroblastoma LAN-1 cells, chronic myelogenous leukemia K562 cells and acute myeloid leukemia NOMO-1 cells (**Figure 2E**). This is of note, as donors were not specifically selected for only high Vδ1 cell purity, but rather represented a range of γδT-subset compositions (a range of harvested αβTCR/CD56-depleted PBMC-derived products is illustrated in **Figure 2F**). This suggests that maximal Vδ1 cell purity does not uniquely determine the cytotoxic potential of the OKT-3/IL-15-expanded product.

**Figure 3:**
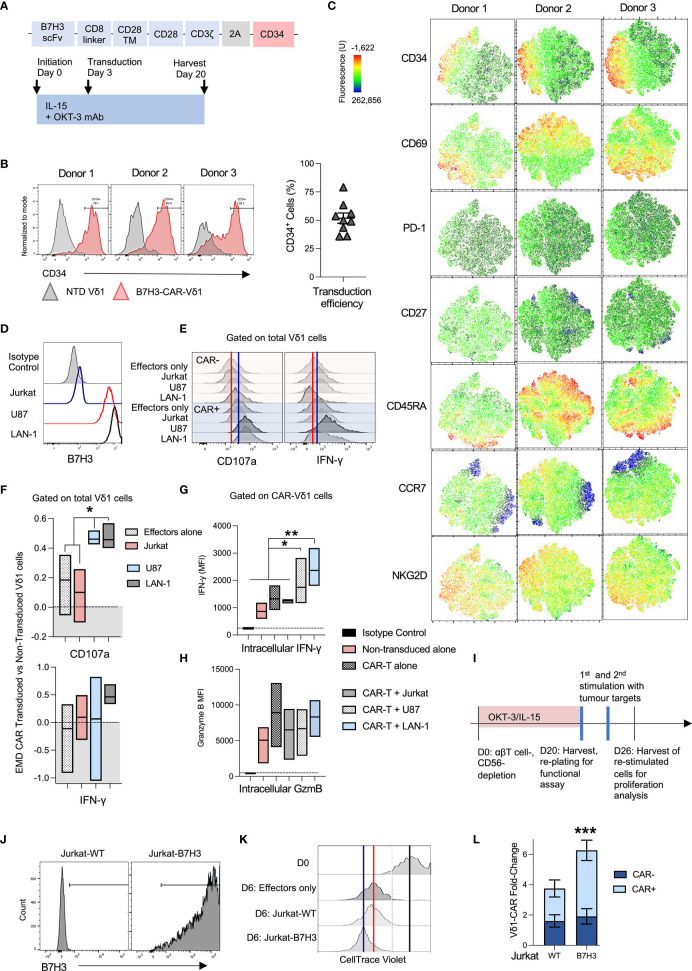
OKT-3/IL-15-expanded Vδ1 cells are readily transducible with chimeric antigen receptors (CAR). **(A)** Vδ1 cells were transduced with an SFG retroviral vector encoding a B7H3-28ζ-CAR and a CD34 marker gene separated with a T2A cleavage sequence at day 3 post-activation. **(B)** Three representative donor Vδ1 cell CD34 marker gene expression is shown (left panel), along with a compilation of 9 different donor transduction efficiencies 5 days post-transduction (right panel). **(C)** Vδ1 cell activation and memory markers were characterized in a whole population of CAR-transduced cells. t-SNE data is shown for three individual donor day-20 harvested PBMC, gated on live Vδ1 cells. **(D)** A panel of B7H3-positive and negative target cell lines was selected. **(E)** Vδ1 cell accumulation of intracellular IFN-γ and cell surface CD107a was measured after overnight co-culture with targets followed by a 4h culture in monensin-supplemented media. Marker accumulation was measured in CAR-transduced and non-transduced Vδ1 cells. Shown are representative histograms of marker expression from a representative donor. The red line indicates median fluorescence intensity (MFI) of CAR-negative effectors alone, while the blue line indicates MFI of CAR-positive effectors alone. **(F)** IFN-γ and CD107a expression histogram data from 3 separate donors was converted to earth mover’s distance (EMD) values that compared marker expression between CAR-transduced *versus* non-transduced cells. A score of ‘0.0’ indicates no difference and is indicated by the dotted line (N=3; mean and distribution indicated). **(G)** The same data was analyzed using MFI measurements, compared in stratified CAR-positive (CD34+) and non-transduced (CD34-) Vδ1 cells in the same culture (N=3; mean and distribution indicated; statistical significance ascertained using one-way ANOVA). **(H)** Vδ1 cells in the same culture were similarly analyzed for intracellular granzyme B levels (N=3; mean and distribution indicated). **(I)** To test expanded CAR-Vδ1 persistence and proliferation, expanded cells were harvested and challenged twice at a 1:1 E:T ratio with **(J)** irradiated B7H3 antigen-positive and negative Jurkat targets. **(K)** CAR-Vδ1 CellTrace Violet dye dilution was measured after 6 day co-culture. One representative donor matched data is shown. The black line indicates the edge of undiluted dye at day 0 of the assay, the red line indicates dye MFI of CAR-Vδ1 only at day 6 indicative of background proliferation, while the blue line indicates dye MFI of CAR-Vδ1 in co-culture with antigen-positive targets. **(L)** To account for ongoing background proliferation, Vδ1 cells were counted pre and post-co-culture using flow cytometric counting beads, and Vδ1 fold-change was normalized to effectors alone (N=3; mean +/- SEM; statistical significance was ascertained using a two-way ANOVA with Sidak’s multiple comparison). * means *P* < 0.05; ** means *P* ≤ 0.01; *** means *P* ≤ 0.001.

### OKT-3/IL-15-Expanded Vδ1 Cells Are Readily Transducible With Chimeric Antigen Receptors (CAR)

To assess the suitability of this expansion protocol for generating genetically-modified immunotherapeutics, we evaluated Vδ1 cell retroviral transduction with an anti-B7H3 2^nd^ generation 28ζ chimeric antigen receptor (CAR) (**Figure 3A**). A consistent ~50% transduction efficiency (ranging from 35.8% - 79.1%) was achieved transducing nine different donors in three experimental runs (**Figure 3B**).

We queried the impact of viral transduction with an ITAM-containing CAR on OKT-3/IL-15 Vδ1 cell product by comparing expression of a range of memory, exhaustion and functional markers within the transduced cell population. Cells were transduced on day 3 following initiation, and thereafter expanded for an additional 17 days until harvest at day 20. Anti-CD34 staining was used to detect expression of the RQR8 CAR marker gene (26) in the transduced cell product. Unexpectedly, none of the activation, memory or exhaustion markers we tested mapped neatly onto CAR(CD34+)-Vδ1 cells (**Figure 3C**). The closest matches were increased expression of NKG2D and a dim but consistent association of CD34 with PD-1 expression in CAR-Vδ1 compared to unmodified Vδ1 cells. Curiously, there was little association between CD69 and CD34 in any of the donors tested, suggesting that the Vδ1 cell product was highly activated regardless of CAR expression. The most notable difference between CAR-transduced Vδ1 cells as a whole compared to OKT-3/IL-15 Vδ1 cells that were never exposed to retrovirus (**Figure 2A**) was the downregulation of CD27 in virus-exposed compared to non-exposed cells. Most other queried markers were similar between both populations.

CAR-Vδ1 were then tested against a range of antigen-positive and negative hematological and solid tumor targets: B7H3-negative Jurkat cells, and B7H3-positive cell lines U87 (originating from glioblastoma) and LAN-1 cells (**Figure 3D**). Intracellular IFN-γ and cytotoxic degranulation cell surface marker CD107a accumulation was compared in CAR-transduced *versus* unmodified Vδ1 cells using flow cytometry (**Figure 3E**). The red line in **Figure 3E** indicates marker median fluorescence intensity (MFI) of unmodified target-free Vδ1 cells, while the blue line indicates the MFI of target-free CAR-Vδ1 cells. These measures are included to account for the innate, B7H3-independent reactivity of Vδ1 cells, as well as potential baseline activation mediated by CAR-transduction. Differences between histograms were quantified using the statistical analysis tool Earth Mover’s Distance (EMD), which quantifies the dissimilarity between two dimensional distributions whilst respecting the single-cell nature of the dataset; a greater value of EMD indicates greater difference (see methods). EMD scores were generated measuring the difference between bulk Vδ1 cell IFN-γ or CD107a accumulation when either unmodified or transduced with a B7H3-28ζ-CAR and challenged with different tumor targets. An EMD score of 0 indicates no relative change between transduced and non-transduced Vδ1 cells. Interestingly, while CAR-Vδ1 CD107a-mediated cytotoxic degranulation was significantly higher upon challenge with antigen-positive U87 and LAN-1 targets than without challenge or challenge with antigen-negative Jurkat cells, IFN-γ production was less consistently impacted by the presence of target antigen and highly variable on a donor-donor basis (**Figure 3F**). This inconsistency was caused not by the inability of CAR engagement to mediate IFN-γ production, but rather high innate and non-CAR-dependent IFN-γ production in some of the donors. All donor Vδ1 cells demonstrated intracellular IFN-γ with and without CAR transduction. A significant, antigen-dependent upregulation of intracellular IFN-γ could be observed when gating on specifically CAR-positive Vδ1 cells, rather than bulk Vδ1 cells in culture (**Figure 3G**). IFN-γ production correlated positively with Vδ1 cell CAR marker gene, CD34, expression when challenged with antigen-positive but not negative targets (**Supplementary Figure 5**). Consistent with a high and sustained cytotoxic potential, granzyme B levels were at least as high or higher in matched unmodified Vδ1 cells compared to CAR-Vδ1 cells before and after challenge with targets (**Figure 3H**).

To test proliferative and persistence capacity, CAR-Vδ1 were harvested post-expansion, plated with no exogenous cytokine and challenged twice at a 1:1 E:T ratio at three day intervals with irradiated B7H3-negative Jurkat wild type cells (Jurkat-WT) or isogenic Jurkat cells transduced to express B7H3 (Jurkat-B7H3) (**Figures 3I, J**). Expansion was monitored *via* dilution of CellTrace Violet proliferation dye, as well as cell counts performed using Precision Count beads and flow cytometry. While all CAR-Vδ1 were highly activated and continued low-grade proliferation after re-plating, more proliferation was seen upon challenge with Jurkat-B7H3 compared to no targets or Jurkat-WT (**Figure 3K**). The black line in **Figure 3K** indicates the CellTrace Violet MFI of Vδ1 cells at plating, the red line of effectors only after 6 days in culture, and the blue line – of effectors co-cultured with antigen-positive targets. Normalized to effectors only, Vδ1 cells expanded more when expressing a CAR but only in response to antigen-positive Jurkat cells (**Figure 3L**). Unmodified Vδ1 cells expanded ~2-fold over target-free matched effectors, likewise CAR-Vδ1 in response to Jurkat-WT. In response to Jurkat-B7H3, meanwhile CAR-Vδ1 cells expanded 4-fold.

## Discussion

We set out to develop a single-step, GMP-compatible CAR-Vδ1 cell expansion and transduction protocol that utilizes standard T cell therapy expansion reagents already employed in the CAR-T field. To that end, we focused on pan-T cell stimulating anti-CD3 mAb, clone OKT-3, and the classic T cell cytokine expansion milieu of IL-2 and IL-15. While OKT-3 with IL-2 failed to support sufficient Vδ1 cell expansion, OKT-3 with IL-15 led to substantial Vδ1 cell expansion that was further boosted by depletion of CD56-positive cells. The additive effect of CD56-positive cell depletion was likely at least in part mediated by decreasing competition for IL-15 from CD56-expressing PBMC, such as NK cells. Given the pan-T cell stimulatory nature of both OKT-3 and IL-15, stringent αβT cell depletion prior to initiation was obligate for achieving Vδ1 cell yield and purity. αβTCR/CD56-depleted OKT-3/IL-15-stimulated Vδ1 cells were highly tumor-reactive in their own right and amenable to transduction to high efficiency with a second generation B7H3-28ζ CAR using standard retroviral protocols. CAR-Vδ1 cells retained innate tumor responsiveness while also engaging in CAR-directed reactivity. Upon challenge with targets, B7H3-28ζ-Vδ1 exhibited antigen-specific persistence, cytotoxicity and IFN-γ production. Taken together, we have described a fully GMP-compatible CAR-Vδ1 manufacturing protocol that utilizes reagents and processes well practiced in the CAR-T field.

We further examined the additional purification of Vδ1 cell product with Vδ2 cell depletion. Vδ2 cells were effectively removable using anti-Vδ2TCR mAb clone B6 conjugated to biotion, magnetically removed with anti-biotin microbeads. These depletions could be successfully carried out at initiation of culture with a triple αβTCR/CD56/Vδ2 depletion, midway through depletion at culture split or at harvest. We reserve judgement as to the best approach in this instance, or whether Vδ2 cell depletion is required at all. We hypothesize that an ultra-pure Vδ1 cell product may not exhibit improved efficacy over a product that contains other γδT cell populations. Though, this warrants substantial further investigation with a range of donors. Indeed, it will be difficult to assess optimal product composition until such products are tested clinically. As the debate for “which γδT cell subset is best?” pervades the immunotherapy field, we expect that only clinical testing and conscientious and scientific clinical trial design will shed light on these questions. Ultimately, it may be that no single subset is superior, but rather that a correct balance of the different subsets is optimal for anti-cancer targeting.

In developing the optimized protocol described herein we used the “DOT protocol” cytokine cocktail described by Almeida and colleagues (23) as a comparator, investigating whether we can design a simplified process. Vδ1 cell yield and phenotype were broadly similar between cells expanded with either OKT3/IL-15 or the “DOT protocol” cocktail of cytokines. There are four main modifications in our process compared to the published “DOT protocol”: (1) a simultaneous αβTCR- and CD56-bead depletion step replaces the αβTCR-depletion only, (2) the “DOT protocol” employs a second OKT-3-based CD3 positive selection step while our protocol adds OKT-3 to the depleted product without the need for a second selection step, 3) the multi-cytokine cocktail of the “DOT protocol” is replaced by IL-15 alone, 4) a second OKT-3 stimulation in the “DOT protocol” midway through expansion in omitted in our protocol. Together these changes represent a considerable simplification of the Vδ1 cell expansion process, and a reduction in cost. It was beyond the scope of the current study to perform a detailed side-by-side comparison in terms of *in vitro* and *in vivo* effector function. Further studies are warranted to compare the long-term effector function between these approaches.

We anticipate an increase of pre-clinical and clinical gene-engineered Vδ1 cell investigations for oncology indications in what is a rapidly evolving immunotherapeutic landscape. With the clinical success of canonical autologous CAR-αβT for a range of B cell malignancies, a role may be carved out for allogeneic non-canonical cell therapies. This includes γδT cells of Vδ1 and Vγ9Vδ2 subsets, as well as NK cells, for the targeting of solid tumor indications and CAR-αβT refractory hematological cancers. Allogeneic approaches of this type may further play an important role in democratizing access to a new generation of gene-engineered cell therapy drugs that can be manufactured in bulk from healthy donor material, with accompanying reductions in price and supply chain complexity, as well as possible improvement in product clinical efficacy.

An important area of ongoing research remains the identification of ‘optimal’ donors for allogeneic cell therapy products. It remains unclear whether high product yield during manufacture is a sure indicator of maximum therapeutic performance, or as recent data from the CAR-αβT field suggests (5) – that cell ‘quality’, including memory and exhaustion status, is a more predictive metric than quantity. The elucidation of the factors that govern γδT cell product ‘quality’ will be crucial to sustained clinical success. Vδ1 cells expanded with this one-step IL-15/OKT-3 process expressed high CD27 and CD45RA, in a pattern that is consistent with naïve and central memory in αβT cells and was diminished upon transduction with B7H3-28ζ-CAR. It is unclear whether this marker expression profile correlates with αβT-like memory phenotypes in Vδ1 cells. Indeed, relatively little is known of γδT cell memory, and less still how such cell surface marker phenotypes correlate with anti-tumor functionality. Expanded and CAR-transduced Vδ1 cells weakly upregulated PD-1 and strongly upregulated TIM-3 ‘exhaustion’ markers, the significance of which on γδT cells is little understood. It is unclear whether their presence is indicative of true T cell exhaustion, activation or something other still.

These properties may further vary between the types of indications targeted and gene engineering applied. Intelligent clinical trial design and study of adoptively transferred γδT cells pre- and post-infusion into patients will be crucial in elucidating the specific qualities of cells that confer the greatest therapeutic benefit.

## Data Availability Statement

The original contributions presented in the study are included in the article/**Supplementary Material**, further inquiries can be directed to the corresponding author/s.

## Ethics Statement

Ethical approval was granted by the UCL UK research ethics committee under IRAS project ID-154668.

## Author Contributions

MB and JA designed the experiments and wrote the manuscript. GF, CA, MF, and SD performed the data-generating experiments for this paper. JF provided data analysis. KC co-supervised CA. All authors contributed to the article and approved the submitted version.

## Funding

This work was supported by the following research grants and awards: TC BioPharm Ltd studentship to GF (SR16A33), Cancer Research UK-City of London Centre Clinical Academic Training Programme Award [C355/A28852] to CA, Stand up to Cancer/Cancer Research UK Pediatric Cancer New Discoveries Challenge (RT6188) to MB, Debbie Fund award to KC, Children with Cancer UK (15-502), Great Ormond Street Charity Infrastructure award (VS0118). JA is supported by the NIHR Great Ormond Street Biomedical Research centre award.

## Conflict of Interest

MF and SD are employed by TC BioPharm Ltd. JA and JF are both inventors on a patent pertaining to CCRs in γδT cells, which was licensed to TC Biopharm (WO/2016/174461). JF has undertaken paid consultancy work for TC BioPharm Ltd. MB was previously employed by TC BioPharm Ltd. JA holds founder stock in Autolus Ltd and share options in TC BioPharm Ltd and holds patents in CAR-T technology. JA received consultancy payments from TC-Biopharm between 2018-2021.

The remaining authors declare that the research was conducted in the absence of any commercial or financial relationships that could be construed as a potential conflict of interest.

## Publisher’s Note

All claims expressed in this article are solely those of the authors and do not necessarily represent those of their affiliated organizations, or those of the publisher, the editors and the reviewers. Any product that may be evaluated in this article, or claim that may be made by its manufacturer, is not guaranteed or endorsed by the publisher.

## References

[1] Lopes N McIntyre C Martin S Raverdeau M Sumaria N Kohlgruber AC. Distinct Metabolic Programs Established in the Thymus Control Effector Functions of γδ T Cell Subsets in Tumor Microenvironments. Nat Immunol (2021) 22:179–92. doi: 10.1038/s41590-020-00848-3 PMC7610600 33462452

[2] Barisa M Kramer AM Majani Y Moulding D Saraiva L Bajaj-Elliott M. Coli Promotes Human Vγ9vδ2 T Cell Transition From Cytokine-Producing Bactericidal Effectors to Professional Phagocytic Killers in a TCR-Dependent Manner. Sci Rep-uk (2017) 7:2805. doi: 10.1038/s41598-017-02886-8 PMC5459831 28584241

[3] Willcox BE Willcox CR. γδ TCR Ligands: The Quest to Solve a 500-Million-Year-Old Mystery. Nat Immunol (2019) 20:121–8. doi: 10.1038/s41590-018-0304-y 30664765

[4] Fisher J Sharma R Don D Barisa M Hurtado M Abramowski P. Engineering γδt Cells Limits Tonic Signaling Associated With Chimeric Antigen Receptors. Sci Signal (2019) 12:eaax1872. doi: 10.1126/scisignal.aax1872 31506382 PMC7055420

[5] Gavriil A Barisa M Halliwell E Anderson J. Engineering Solutions for Mitigation of Chimeric Antigen Receptor T-Cell Dysfunction. Cancers (2020) 12:2326. doi: 10.3390/cancers12082326 PMC7463974 32824734

[6] Fowler D Nattress C Navarrete AS Barisa M Fisher J. Payload Delivery: Engineering Immune Cells to Disrupt the Tumour Microenvironment. Cancers (2021) 13:6000. doi: 10.3390/cancers13236000 34885108 PMC8657158

[7] Barisa M Fowler D Fisher J. Interplay Between γδt-Cell Metabolism and Tumour Microenvironment Offers Opportunities for Therapeutic Intervention. Immunometabolism (2021) 3:210026. doi: 10.20900/immunometab20210026 34394978 PMC7611484

[8] Gentles AJ Newman AM Liu CL Bratman SV Feng W Kim D. The Prognostic Landscape of Genes and Infiltrating Immune Cells Across Human Cancers. Nat Med (2015) 21:938–45. doi: 10.1038/nm.3909 26193342 PMC4852857

[9] Park JH Kim H-J Kim CW Kim HC Jung Y Lee H-S. Tumor Hypoxia Represses γδ T Cell-Mediated Antitumor Immunity Against Brain Tumors. Nat Immunol (2021) 22:336–46. doi: 10.1038/s41590-020-00860-7 33574616

[10] Davey MS Willcox CR Joyce SP Ladell K Kasatskaya SA McLaren JE. Clonal Selection in the Human Vδ1 T Cell Repertoire Indicates γδ TCR-Dependent Adaptive Immune Surveillance. Nat Commun (2017) 8:14760. doi: 10.1038/ncomms14760 28248310 PMC5337994

[11] Fisher JP Heuijerjans J Yan M Gustafsson K Anderson J. γδ T Cells for Cancer Immunotherapy: A Systematic Review of Clinical Trials. Oncoimmunology (2014) 3:e27572. doi: 10.4161/onci.27572 24734216 PMC3984269

[12] Hurtado MO Wolbert J Fisher J Flutter B Stafford S Barton J. Tumor Infiltrating Lymphocytes Expanded From Pediatric Neuroblastoma Display Heterogeneity of Phenotype and Function. PLoS One (2019) 14:e0216373. doi: 10.1371/journal.pone.0216373 31398192 PMC6688820

[13] Landin AM Cox C Yu B Bejanyan N Davila M Kelley L. Expansion and Enrichment of Gamma-Delta (γδ) T Cells From Apheresed Human Product. J Vis Exp Jove (2021) 175:e62622. doi: 10.3791/62622 34633380

[14] Wang S. Expansion of Gamma Delta T Cells - A Short Review on Bisphosphonate and K562-Based Methods. J Immunol Sci (2018) 2:6–12. doi: 10.29245/2578-3009/2018/3.1133

[15] Siegers GM Dhamko H Wang X-H Mathieson AM Kosaka Y Felizardo TC. Human Vδ1 γδ T Cells Expanded From Peripheral Blood Exhibit Specific Cytotoxicity Against B-Cell Chronic Lymphocytic Leukemia-Derived Cells. Cytotherapy (2011) 13:753–64. doi: 10.3109/14653249.2011.553595 21314241

[16] Knight A Mackinnon S Lowdell MW. Human Vdelta1 Gamma-Delta T Cells Exert Potent Specific Cytotoxicity Against Primary Multiple Myeloma Cells. Cytotherapy (2012) 14:1110–8. doi: 10.3109/14653249.2012.700766 22800570

[17] Correia DV Fogli M Hudspeth K da SMG Mavilio D Silva-Santos B. Differentiation of Human Peripheral Blood Vδ1+ T Cells Expressing the Natural Cytotoxicity Receptor NKp30 for Recognition of Lymphoid Leukemia Cells. Blood (2011) 118:992–1001. doi: 10.1182/blood-2011-02-339135 21633088

[18] Wu D Wu P Wu X Ye J Wang Z Zhao S. Ex Vivo Expanded Human Circulating Vδ1 γδt Cells Exhibit Favorable Therapeutic Potential for Colon Cancer. Oncoimmunology (2015) 4:e992749. doi: 10.4161/2162402x.2014.992749 25949914 PMC4404819

[19] Chien Y Konigshofer Y. Antigen Recognition by Gammadelta T Cells. Immunol Rev (2007) 215:46–58. doi: 10.1111/j.1600-065X.2006.00470.x 17291278

[20] Butturini A Bortin MM Seeger RC Gale RP. Graft-Vs-Leukemia Following Bone Marrow Transplantation: A Model of Immunotherapy in Man. Prog Clin Biol Res (1987) 244:371–90. 3310000

[21] Lamb LS Gee AP Hazlett LJ Musk P Parrish RS O’Hanlon TP. Influence of T Cell Depletion Method on Circulating γδ T Cell Reconstitution and Potential Role in the Graft-Versus-Leukemia Effect. Cytotherapy (1999) 1:7–19. doi: 10.1080/0032472031000141295 19746645

[22] Godder KT Henslee-Downey PJ Mehta J Park BS Chiang K-Y Abhyankar S. Long Term Disease-Free Survival in Acute Leukemia Patients Recovering With Increased γδ T Cells After Partially Mismatched Related Donor Bone Marrow Transplantation. Bone Marrow Transpl (2007) 39:751–7. doi: 10.1038/sj.bmt.1705650 17450185

[23] Almeida AR Correia DV Fernandes-Platzgummer A da SCL da SMG DR A. Delta One T Cells for Immunotherapy of Chronic Lymphocytic Leukemia: Clinical-Grade Expansion/Differentiation and Preclinical Proof of Concept. Am Assoc Cancer Res (2016) 22:5795–804. doi: 10.1158/1078-0432.ccr-16-0597 27307596

[24] Orlova DY Zimmerman N Meehan S Meehan C Waters J Ghosn EEB. Earth Mover’s Distance (EMD): A True Metric for Comparing Biomarker Expression Levels in Cell Populations. PLoS One (2016) 11:e0151859. doi: 10.1371/journal.pone.0151859 27008164 PMC4805242

[25] Fisher JPH Yan M Heuijerjans J Carter L Abolhassani A Frosch J. Neuroblastoma Killing Properties of Vδ2 and Vδ2-Negative γδt Cells Following Expansion by Artificial Antigen-Presenting Cells. Clin Cancer Res (2014) 20:5720–32. doi: 10.1158/1078-0432.ccr-13-3464 PMC4445920 24893631

[26] Philip B Kokalaki E Mekkaoui L Thomas S Straathof K Flutter B. A Highly Compact Epitope-Based Marker/Suicide Gene for Easier and Safer T-Cell Therapy. Blood (2014) 124:1277–87. doi: 10.1182/blood-2014-01-545020 24970931

