# Supplementary material for: A Simple and Robust Single-Step Method for CAR-Vδ1 γδT Cell Expansion and Transduction for Cancer Immunotherapy
Source: Front Immunol. 2022 May 31;13:863155. doi: 10.3389/fimmu.2022.863155 (PMC9197253; doi:10.3389/fimmu.2022.863155)
Supplement: Supplementary file 1 [file DataSheet_1.pdf]

Supplementary Material

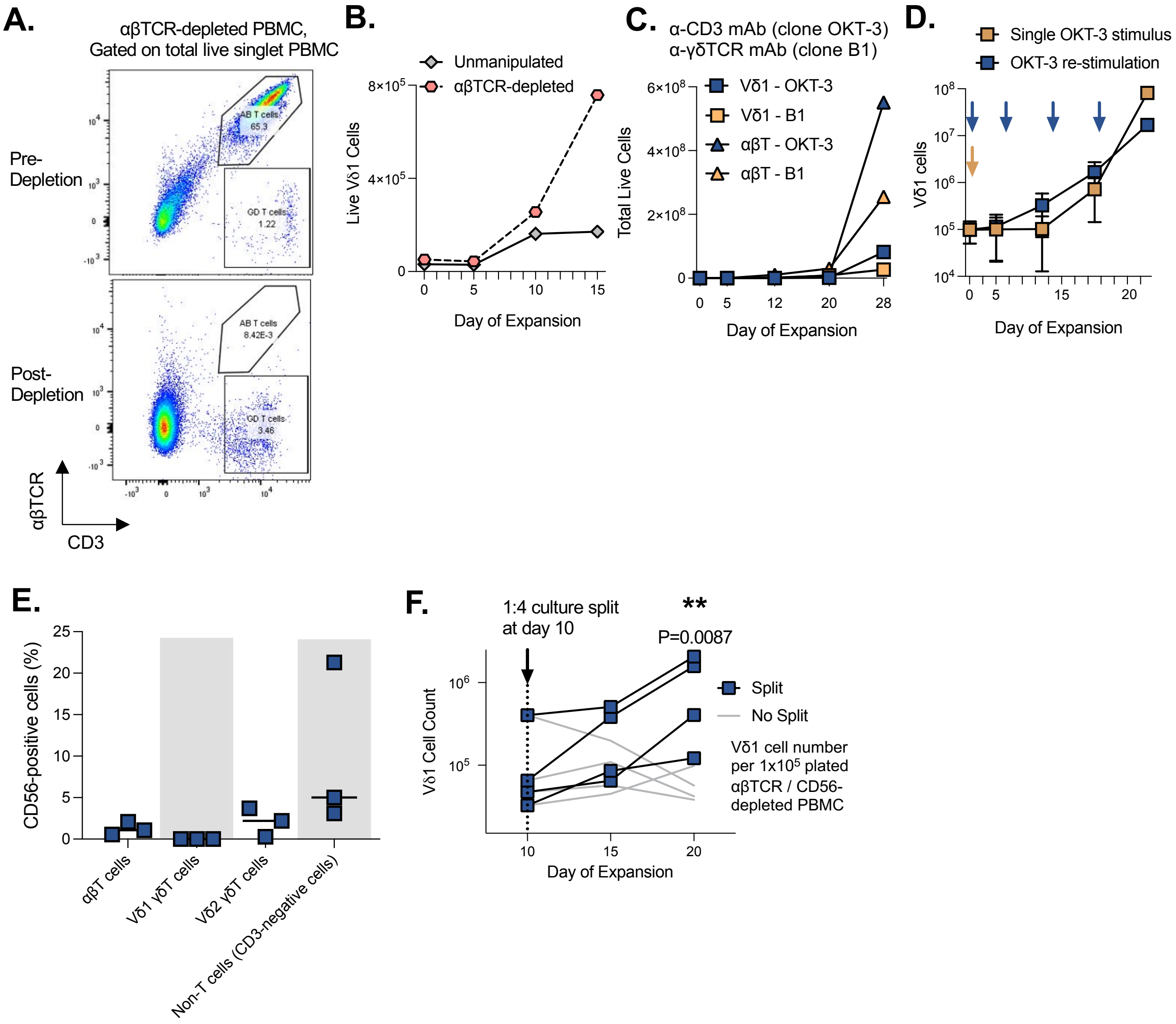

Supplementary Figure 1. Optimising Vδ1 cell expansion with clone OKT-3 anti-CD3 mAb. (**A**) Freshly-isolated PBMC were depleted of αβT cells. Shown are representative dot plots of PBMC composition pre- and post-depletion, gated on live singlet lymphocytes. (**B**) Vδ1 cells were expanded using the 'DOT' cocktail of cytokines from either whole PBMC or PBMC first depleted of αβT cells (N=1). (**C**) Matched PBMC were stimulated with the 'DOT' cocktail of cytokines and either anti-

γδTCR mAb clone B1 or OKT-3. αβT cells and Vδ1 cells were counted. (**D**) Matched PBMC were stimulated with the 'DOT' cocktail of cytokines and either a single dose of OKT-3 at initiation (indicated by yellow arrow) or multiple doses (indicated by blue arrows). Vδ1 cells were counted (N=2). (**E**) CD56 expression was characterized in three separate donor freshly-isolated PBMC. Each dot indicates a separate donor. A mean is indicated with a black line. (**F**) αβTCR/CD56-depleted PBMC were expanded with OKT-3/IL-15 from 4 donors until day 10, and then either split 1:4 or left undisturbed in culture. Vδ1 cells were counted (N=4; statistical significance was ascertained using a matched two-way ANOVA with Sidak's multiple comparison test).

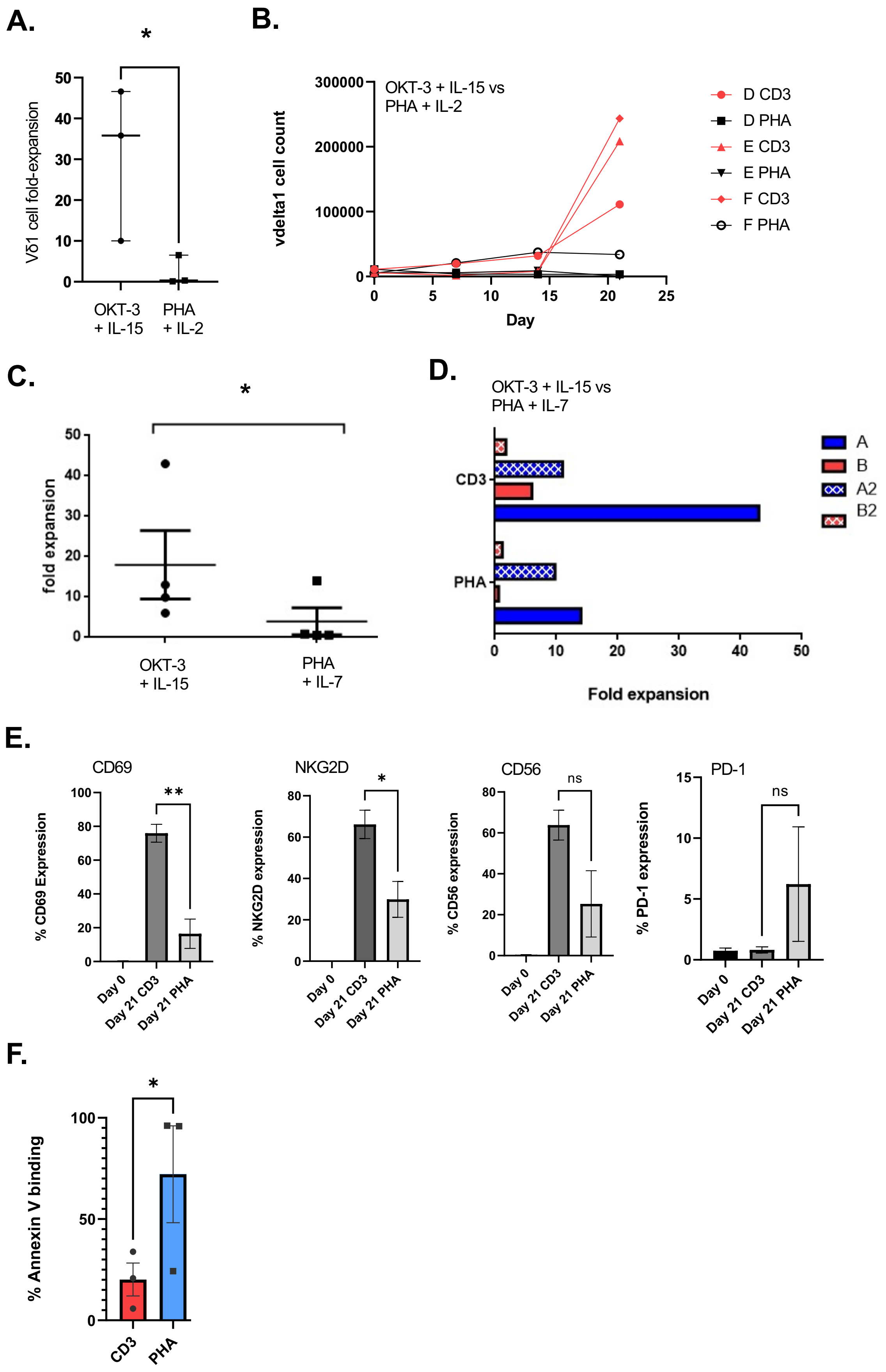

A.
*
Vδ1 cell fold-expansion
OKT-3 + IL-15
PHA + IL-2
B.
OKT-3 + IL-15 vs PHA + IL-2
vdelta1 cell count
Day
D CD3
D PHA
E CD3
E PHA
F CD3
F PHA
C.
*
fold expansion
OKT-3 + IL-15
PHA + IL-7
D.
OKT-3 + IL-15 vs PHA + IL-7
CD3
PHA
A
B
A2
B2
Fold expansion
E.
CD69
% CD69 Expression
**
NKG2D
% NKG2D expression
*
CD56
% CD56 expression
ns
PD-1
% PD-1 expression
ns
Day 0
Day 21 CD3
Day 21 PHA
F.
*
% Annexin V binding
CD3
PHA

Supplementary Figure 2. Vδ1 cell expansion using OKT-3 *versus* PHA. Vδ1 cell fold-expansion following three-week culture following a single stimulation at initiation with either OKT-3 or phytohaemagglutinin (PHA). (**A**) αβTCR- and CD56-depleted PBMC were stimulated with OKT-3 / IL-15 or PHA / IL-2 (N=3; median with distribution). (**B**) Individual donor trajectories (D, E, F) are shown (N=3). (**C**) αβTCR- and CD56-depleted PBMC were stimulated with OKT-3 / IL-15 or PHA / IL-7 (N=3; mean +/- SEM). (**D**) Individual donor (A, A2, B, B2) day 20 Vδ1 cell fold-expansion are shown (N=3). (**E**) Day 20-expanded OKT-3 / IL-15 and PHA / IL-7 activation and (**F**) apoptotic marker expression was compared on freshly-expanded and expanded Vδ1 cells (N=3; mean +/- SEM; statistical significance was assessed using one-way ANNOVA).

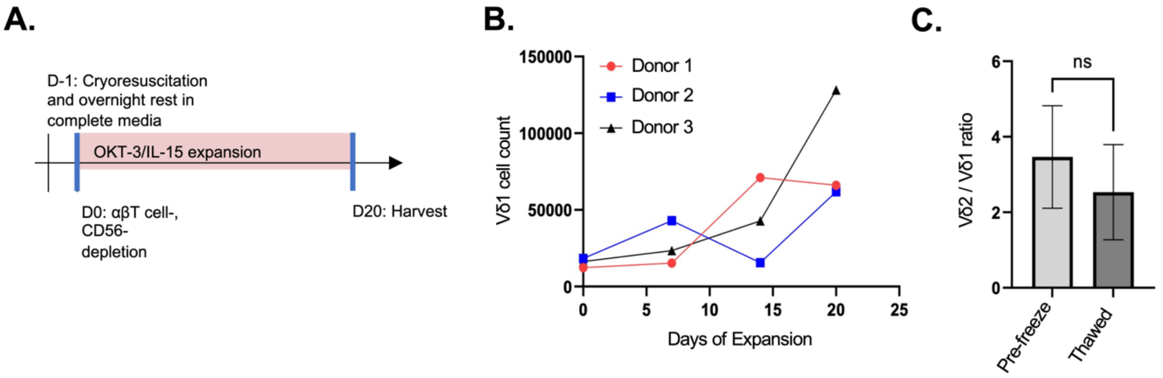

Supplementary Figure 3. Vδ1 cell expansion from cryopreserved PBMC. (**A**) PBMC from three different donors were cryopreserved, thawed, rested overnight and then depleted of αβT and CD56-positive cells, and stimulated with OKT-3 and IL-15. (**B**) Vδ1 cells from this expansion were counted using flow cytometry. (**C**) Vδ1/ Vδ2 cell ratio as ascertained by flow cytometry was compared in PBMC pre- and post-cryopreservation (N=3). Statistical significance was ascertained using a paired T-test.

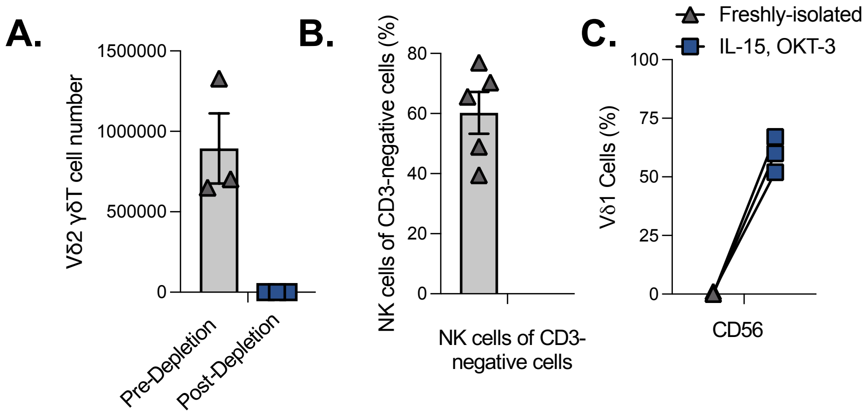

Supplementary Figure 4. Vδ1 cell manufacture depletions and product composition. (**A**) Freshly-isolated PBMC from three different donors were αβTCR / CD56 / Vδ2 triple-depleted. Vδ2 cells were counted pre- and post-depletion. (**B**) Expanded T cells from αβTCR / CD56 substrate were depleted on day 20 of Vδ2 cells. The NK cell (CD3-CD56+) content of the CD3-negative cells in the purified product was measured (N=5). (**C**) CD56 expression was measured on freshly-isolated and day 20- OKT-3/IL-15 expanded Vδ1 cells (N=3).

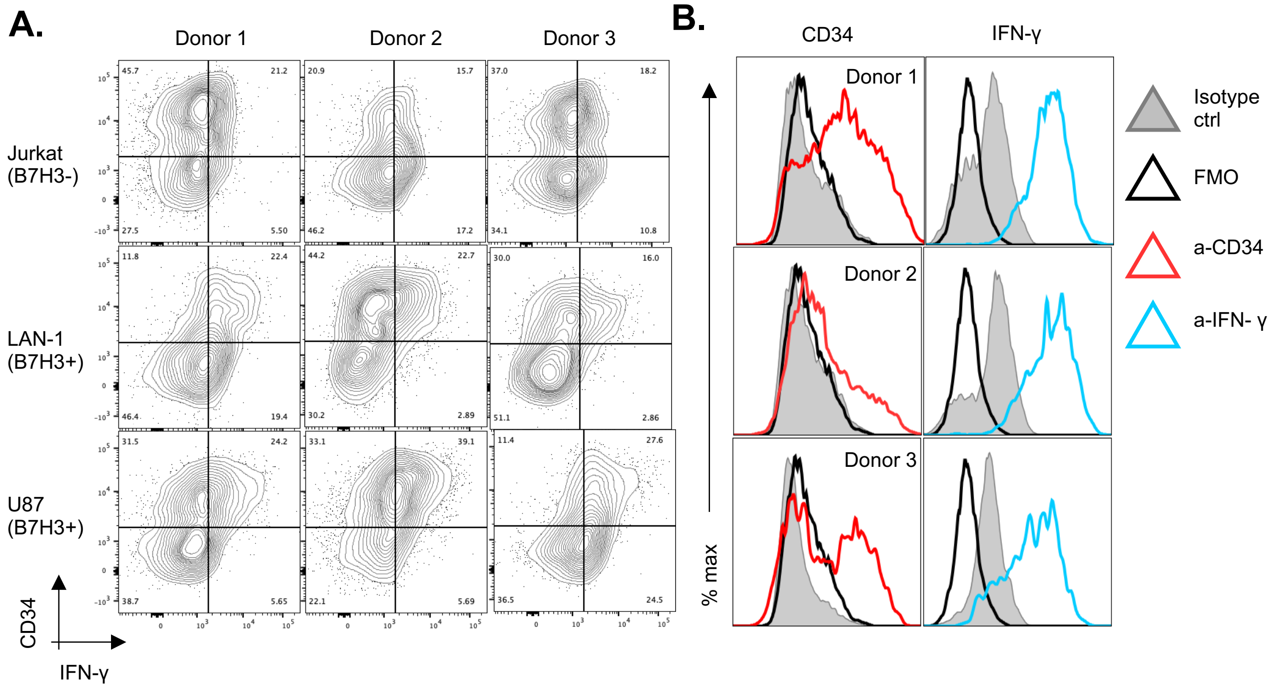

Supplementary Figure 5. CAR-Vδ1 cell intracellular IFN-γ accumulation. (A) CAR-transduced cultures of expanded Vδ1 cells were challenged with either antigen-positive or negative targets at a 1:1 E:T ratio overnight, before a 4h culture in monensin. Intracellular IFN-γ accumulation was assessed using flow cytometry. Shown is three different donor Vδ1 cell expression of IFN-γ *versus* transduction marker gene CD34. (B) To evaluate antibody binding specificity, three donor CAR-transduced cultures of expanded Vδ1 cells were stained with either isotype control (clone: MOPC-21) antibody or specific antibody within the context of the rest of the staining panel. Shown is representative data for CD34 cell-surface staining and IFN-γ intracellular staining, comparing staining brightness between fluorescence minus one (FMO), isotype control-stained and fully-stained samples.
